# Evidence for coal forest refugia in the seasonally dry Pennsylvanian tropical lowlands of the Illinois Basin, USA

**DOI:** 10.7717/peerj.630

**Published:** 2014-11-04

**Authors:** Cindy V. Looy, Robert A. Stevenson, Thomas B. Van Hoof, Luke Mander

**Affiliations:** 1 Department of Integrative Biology and Museum of Paleontology, University of California, Berkeley, CA, USA; 2 Department of Integrative Biology, University of California, Berkeley, CA, USA; 3 TNO-Geobiology, Utrecht, The Netherlands; 4 College of Life and Environmental Sciences, University of Exeter, Exeter, Devon, UK

**Keywords:** Refugia, Pennsylvanian, Paleoecology, Vegetation reconstruction

## Abstract

The Moscovian plant macroflora at Cottage Grove southeastern Illinois, USA, is a key example of Pennsylvanian (323–299 Million years ago) dryland vegetation. There is currently no palynological data from the same stratigraphic horizons as the plant macrofossils, leaves and other vegetative and reproductive structures, at this locality. Consequently, reconstructions of the standing vegetation at Cottage Grove from these sediments lack the complementary information and a more regional perspective that can be provided by sporomorphs (prepollen, pollen, megaspores and spores). In order to provide this, we have analysed the composition of fossil sporomorph assemblages in two rock samples taken from macrofossil-bearing inter-coal shale at Cottage Grove. Our palynological data differ considerably in composition and in the dominance-diversity profile from the macrofossil vegetation at this locality. Walchian conifers and pteridosperms are common elements in the macroflora, but are absent in the sporomorph assemblages. Reversely, the sporomorph assemblages at Cottage Grove comprise 17 spore taxa (∼16% and ∼63% of the total assemblages) that are known from the lycopsid orders Isoetales, Lepidodendrales and Selaginallales, while Cottage Grove’s macrofloral record fails to capture evidence of a considerable population of coal forest lycopsids. We interpret our results as evidence that the Pennsylvanian dryland glacial landscape at Cottage Grove included fragmented populations of wetland plants living in refugia.

## Introduction

The Pennsylvanian Subperiod of the Carboniferous (323–299 Ma) was characterized by a series of glacial–interglacial cycles that exerted profound control on the distribution of vegetation at this time (Eros et al., 2012). In particular, these cyclic climatic changes resulted in the alternating dominance of wetland and dryland vegetation in the Pennsylvanian tropics (DiMichele, 2014). The wetland vegetation of this time period is represented in the fossil record by the classic Pennsylvanian Coal Forests, which were composed of arborescent lycopsids and, depending on the flooding regime, an understory of marattialean tree ferns, pteridosperms, sphenopsids and cordaitaleans (DiMichele & Phillips, 1994; DiMichele, 2014). In contrast, Pennsylvanian dryland vegetation is recorded by rare gymnosperm-dominated fossil assemblages, which contain drought-tolerant plants such as cordaitaleans, pteridosperms and walchian conifers (Falcon-Lang et al., 2009; Dolby, Falcon-Lang & Gibling, 2011). There is also evidence for the co-existence of wetland and dryland vegetation in at least regional proximity, reflected by fossil remains of dryland plants preserved alongside fossil wetland vegetation. These drier elements are thought to have been transported from upland (extrabasinal *sensu* Pfefferkorn, 1980) areas into wetland basins (e.g., Gastaldo, 1987; Lyons & Darrah, 1989; Falcon-Lang & Bashforth, 2004; Gastaldo & Degges, 2007).

A good example of Pennsylvanian dryland vegetation is the Moscovian age macroflora found at Cottage Grove Mine, Illinois (Falcon-Lang et al., 2009). This flora was discovered in fine siltstone layers closely associated with conglomeratic facies within a shallow channel, laterally equivalent to a calcic vertisol (indicating a seasonally dry climate) just below the Baker Coal in southeastern Illinois, USA (37°46′N, 88°25′W) (Feldman et al., 2005; Falcon-Lang et al., 2009). The Cottage Grove macroflora is composed mostly of cordaitaleans together with pteridosperms, walchian conifers, ferns and sphenopsids, and lacks classic Coal Forest plants such as lycopsids (Falcon-Lang et al., 2009). The small size of the channel (∼250 m wide) and locally derived channel fill indicates that the drainage area was relatively small (Feldman et al., 2005). The plant macrofossils (leaves and other vegetative and reproductive structures) at Cottage Grove are associated with locally derived conglomerate and this, together with information on channel morphology, is interpreted as evidence that the macrofossils represent local vegetation growing on interfluves, close to the depositional environment (Falcon-Lang et al., 2009).

There is currently no palynological data from the same stratigraphic horizons as the plant macrofossils that are preserved at Cottage Grove (Falcon-Lang et al., 2009). Reconstructions of the standing vegetation at this locality therefore lack the complementary data that can be provided by sporomorphs (prepollen, pollen, megaspores and spores) (e.g., Chaloner, 1968; Gastaldo et al., 1998; Jackson & Booth, 2007; Mander, Kürschner & McElwain, 2010). To address this deficit, we have undertaken a palynological investigation of the macrofossil-bearing shale found within the conglomeratic channel facies at Cottage Grove. This shale, and the sampled interval, is important because it was not deposited in association with widespread wetland environments typical of peat forming portions of glacial-interglacial cycles. Our results highlight considerable differences between the macrofossil and sporomorph records at Cottage Grove. Notably, our study reveals a population of 17 species of lycopsids from the orders Isoetales, Lepidodendrales and Selaginallales that flourished at Cottage Grove, but is entirely absent from the macrofossil record at this locality. Consequently, suggestions that Pennsylvanian dryland vegetation at Cottage Grove was dominated by xerophytic plants and devoid of wetland taxa such as lycopsids (Falcon-Lang et al., 2009) are incompatible with our palynological data. We interpret the discrepancy between the macrofossil and sporomorph records at Cottage Grove as evidence that the Pennsylvanian dryland interglacial landscape in this region included fragmented populations of wetland plants living in refugia.

## Materials and Methods

Plant macrofossils at Cottage Grove are preserved in fine-grained siltstones in close association with conglomeratic layers (Falcon-Lang et al., 2009). We have analyzed the palynological composition of two samples from sediments containing a conifer macrofossil that occurred in a layer sandwiched between channel-bottom siltstones and conglomeratic deposits (lithologically unit 6a at the Cottage Grove locality; see Fig. 2A of Falcon-Lang et al., 2009). We have taken a palynological sample from the sediments (Sample 1) in which the conifer macrofossil (USNM 536629; see Fig. 3 of Falcon-Lang et al., 2009) was embedded, and a palynological sample of the counterpart surface (Sample 2). This means that the deposition of the two samples was temporally separated by less than the amount of time it would have taken for the deposited conifer remains to decay. Sample 2 originated from a slightly darker area on the counterpart surface of the same conglomeratic unit.

The sample was bulk macerated using hydrofluoric and hydrochloric acid, and the remaining organic residue was processed at the Laboratory of Palynology and Palaeobotany, Utrecht University, according to palynological techniques (heavy liquid separation and sieving over 15 µm mesh). The samples were not sieved over 250 µm mesh. This will be addressed later. The organic residue was mounted in glycerin jelly. The two samples were screened for identifiable sporomorphs, and a count of 433 (Sample 1) and 300 (Sample 2) sporomorphs was performed at the species level. Additional slides from the two samples were then screened for rare species. Two additional rare taxa were recorded in this process. Taxonomic descriptions by Smith & Butterworth (1967), Ravn (1979) and Ravn (1986) were used for identification. A list of sporomorph taxa, their botanical affinities, and their relative abundances in each of the two samples were compiled (Table 1, Table S1).

**Table 1:** Botanical affinity pollen and spores. Pollen and spores recovered from Cottage Grove plant locality, Cottage Grove Mine, southeast Illinois, together with their botanical affinity. The assignments to major taxonomic groups are based on information in a synthesis (Balme, 1995) and articles covering the same time interval and floral realm (Bashforth et al., 2011; Dimitrova, Cleal & Thomas, 2005; Dimitrova, Cleal & Thomas, 2011; Dolby, Falcon-Lang & Gibling, 2011; Eble, Greb & Williams, 2001; Van Hoof et al., 2013). The plus signs indicate rare, isolated occurrences.

	**Sample 1**	**Sample 2**
**LYCOPSIDS**	**62.6%**	**16.3%**
**Isoetales**		
**Chaloneriaceae**		
*Endosporites globiformis* (Ibrahim) Schopf, Wilson et Bentall 1944	0.5%	0.0%
*Endosporites* sp. Wilson et Coe 1940	0.2%	0.0%
*Radiizonates tenuis* (Loose) Butterworth et Smith, 1964	0.2%	0.0%
*Cristatisporites indignabundus* (Loose) Staplin et Jansonius 1964	0.0%	+
**Lepidodendrales**		
**Diaphorodendraceae**		
*Granasporites medius* (Dybovd et Jachowicz) Ravn et al. 1986	1.4%	12.0%
**Lepidocarpaceae**		
*Cadiospora magna* Kosanke 1950	0.5%	0.3%
*Lycospora brevijuga* Bhardwaj 1957	0.0%	0.7%
*Lycospora brevis* Bhardwaj 1957	2.8%	0.0%
*Lycospora granulata* Kosanke 1950	15.2%	0.0%
*Lycospora parva* Kosanke 1950	0.0%	0.7%
*Lycospora pellucida* (Wicher) Schopf, Wilson et Bentall 1944	9.5%	0.0%
*Lycospora punctata* Kosanke 1950	1.4%	0.0%
*Lycospora pusilla* (Ibrahim) Schopf, Wilson et Bentall 1944	15.9%	0.0%
*Lycospora* sp. (Ibrahim) Schopf, Wilson et Bentall 1944	14.1%	0.0%
**Lepidodendraceae**		
*Crassispora kosankei* Potonié et Kremp 1955	0.0%	2.3%
**Selaginellales**		
**Selaginellaceae**		
*Cirratriradites annulatus* Kosanke 1950	0.5%	0.0%
*Cirratriradites annuliformis* (Kosanke et Brockaw) Kosanke 1950	0.5%	0.3%
**SPHENOPSIDS**	**4.4%**	**5.0%**
**Calamitales**		
**Calamitaceae**		
*Calamospora breviradiata* Kosanke 1950	0.0%	1.3%
*Calamospora microrugosa* (Ibrahim) Schopf, Wilson et Bentall 1944	0.0%	0.7%
*Calamospora parva* Guennel 1958	0.9%	0.7%
**Sphenophyllales**		
*Laevigatosporites minor* Loose 1934	3.0%	1.3%
*Vestispora pseudoreticulata* (Spode) Smith & Butterworth, 1967	0.5%	1.0%
**FERNS**	**16.9%**	**28.3%**
**Filicales**		
*Granulatisporites granulatus* Ibrahim 1933	1.2%	0.7%
**Gleicheniaceae**		
*Triquitrites bransonii* Wilson et Hoffmeister 1956	3.2%	0.0%
*Triquitrites sculptilis* (Balme) Smith & Butterworth, 1967	2.8%	0.7%
**Botryopteridaceae**		
*Microreticulatisporites nobilis* (Wicher) Knox 1950	1.2%	0.3%
**Tedeleaceae**		
*Raistrickia fulva* Artüz 1957	0.0%	0.3%
*Raistrickia irregularis* Kosanke 1950	0.0%	0.3%
*Raistrickia pilosa* Kosanke 1950	0.7%	0.3%
**Marattiales**		
**Marattiaceae**		
*Cyclogranisporites aureus* (Loose) Potonié et Kremp 1955	0.0%	5.3%
*Punctatosporites rotundus* Bhardwaj 1957	0.5%	0.0%
*Laevigatosporites globosus* Schemel 1951	+	0.0%
*Thymospora pseudothiessenii* (Kosanke) Wilson et Venkatachala 1963	3.0%	0.0%
**Psaroniaceae**		
*Latosporites minutus* Bhardwaj 1957	1.2%	0.0%
**Unknown ferns**		
*Deltoidospora levis* (Kosanke) Ravn, 1986	0.0%	0.3%
*Deltoidospora ornata*(Ishchenko) Braman et Hills 1977	0.5%	0.3%
*Deltoidospora priddyi* (Berry) McGregor 1973	0.0%	1.7%
*Deltoidospora tumida* (Butterworth et Williams) Ravn, 1986	0.0%	0.2%
*Deltoidospora* sp. Miner 1935	0.5%	0.0%
*Dictyotriletes mediareticulatus* (Ibrahim) Potonié et Kremp 1955	0.0%	3.3%
*Granulatisporites adnatoides* (Potonié et Kremp) Smith & Butterworth, 1967	0.0%	0.3%
*Leiotriletes tumida* Butterworth et Williams 1958	0.2%	0.0%
*Mooreisporites inusitatus* (Kosanke) Neeves 1958	0.0%	0.3%
*Punctatosporites* sp. Ibrahim 1933	1.2%	0.0%
*Verrucosisporites donarii* Potonié et Kremp 1956	0.2%	9.3%
**Zygopteridales**		
**Zygopteridaceae**		
*Verrucosisporites verrucosis* Ibrahim 1933	0.7%	4.7%
**CORDAITES**	**15.5%**	**50.3%**
**Cordaitanthales**		
**Cordaitanthaceae**		
*Florenites* sp. Schopf, Wilson, et Bentall 1944	1.4%	0.0%
*Florinites florinii* Imgrund 1960	0.9%	1.7%
*Florinites mediapudens* (Loose) Potonié et Kremp 1956	10.6%	37.3%
*Florinites pumicosus* (Ibrahim) Schopf, Wilson et Bentall 1944	2.1%	9.0%
*Florinites visendus* (Ibrahim) Schopf, Wilson et Bentall 1944	0.5%	2.3%
**UNKNOWN AFFINITY**	**0.7%**	**0.0%**
cf. *Punctatisporites*	0.2%	0.0%
*Cheiledonites* sp. Doubinger 1957	0.2%	0.0%
*Cuneisporites rigidus* Ravn, 1979	0.2%	0.0%

## Results

**Figure 1:**
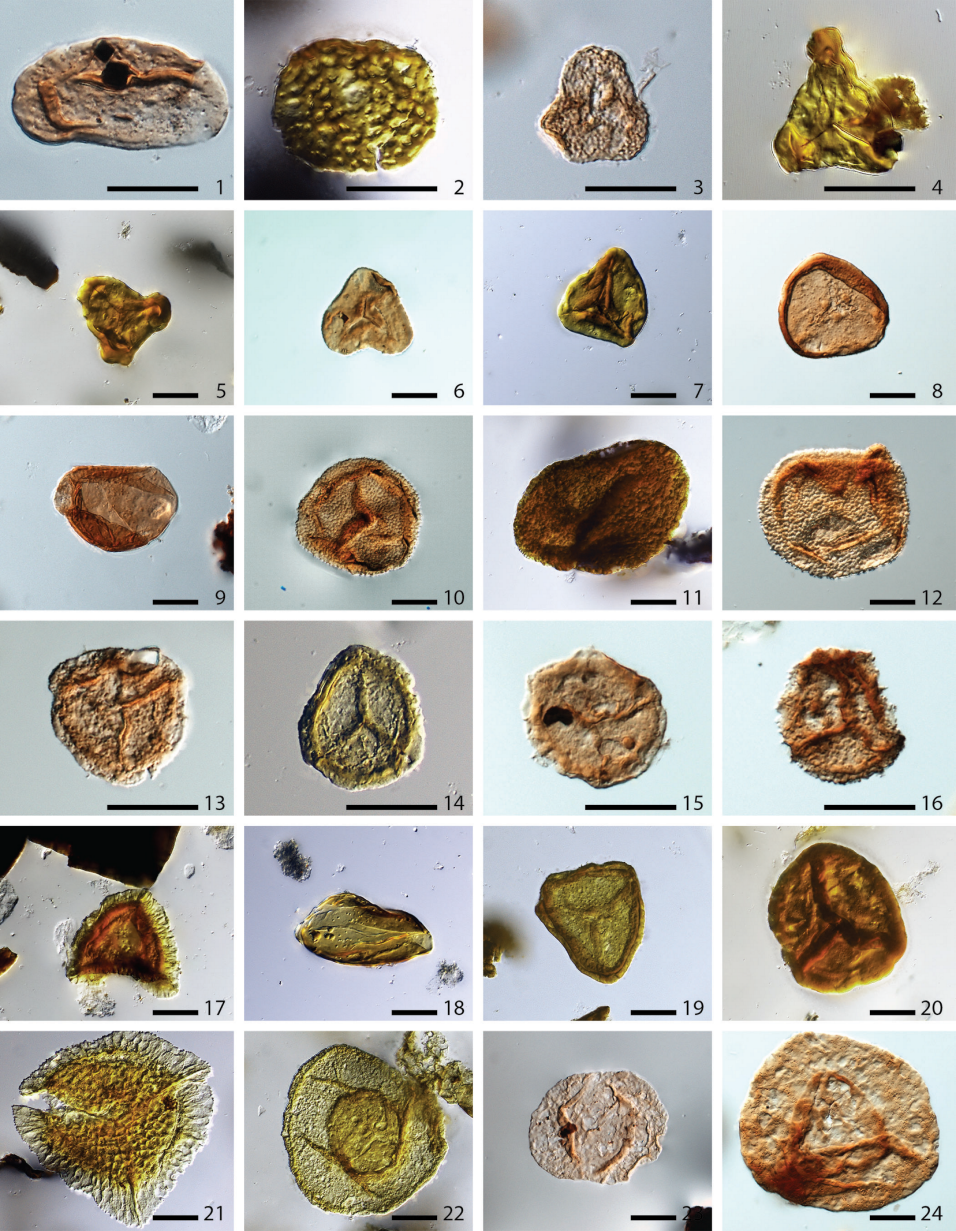
Pollen and spores from Cottage Grove plant locality. Selected pollen and spores from Cottage Grove plant locality, Cottage Grove Mine, southeast Illinois. Specimen names are followed by USNM specimen numbers, slide code, and England Finder graticule coordinates. Scale bars are 20 µm. **1**. *Laevigatosporites minor* (USNM 606400), E36-1. **2**. *Thymospora pseudothiessenii* (USNM 606401), S34-3. **3**. *Granulatisporites granulatus* (USNM 606402), H46-1. **4**. *Triquitrites bransonii* (USNM 606403), Y36. **5**. *Triquitrites sculptilis* (USNM 606404), T45. **6**. *Deltoidospora priddyi* (USNM 606405), T18. **7**. *Deltoidospora ornata* (USNM 606406), K41-4. **8**. *Crassispora kosankei* (USNM 606407), Q39-3. **9**. *Granasporites medius* (USNM 606408), K31. **10**. *Cyclogranisporites aureus* (USNM 606409), R15. **11**. *Verrucosisporites* (USNM 606410), Q34. **12**. *Verrucosisporites verrucosis* (USNM 606411), P30-2. **13**. *Lycospora brevis* (USNM 606412), U44-1. **14**. *Lycospora pusilla* (USNM 606413), Q46-3. **15**. *Lycospora* (USNM 606414), T33-2. **16**. *Lycospora granulata* (USNM 606415), M47-3. **17**. *Radiizonates tenuis* (USNM 606416), U35. **18**. *Cheiledonites* sp. (USNM 606417), P33-2. **19**. *Cuneisporites rigidus* (USNM 606418), J49-2. **20**. *Cadiospora magna* (USNM 606419), Q38-1. **21**. *Cirratriradites annulatus* (USNM 606420), T34-1. **22**. *Endosporites globiformis* (USNM 606421), Q48-1. **23**. *Florinites mediapudens* (USNM 606422), M25-4. **24**. *Florinites pumicosus* (USNM 606423), V41.

The sporomorph assemblage of Sample 1 is dominated by characteristic Middle Pennsylvanian wetland taxa (Table 1). Spores of arborescent lycopsids (e.g., *Cadiospora*, *Lycospora* spp., *Granasporites*) make up the majority of the assemblage (60.8%), with fern spores (16.9%) and cordaitalean prepollen (*Florinites* spp., 15.5%) as the other major components (Fig. 1). Notable occurrences include *Lycospora granulata*, which was produced by the highly specialized wetland arborescent lycopsid *Lepidophloios hallii* (DiMichele, 2014), and represents 15.2% of the total sporomorph assemblage. Sub-arborescent lycopsids are represented by low numbers of *Endosporites, Radiizonates* and *Cristatisporites*. Spores that have been found *in situ* herbaceous lycopods (*Cirratriradites* spp.) are rare elements. Sample 1 also contains the spores of a variety of ferns, including marattialean tree ferns (*Cyclogranisporites*, *Thymospora*, *Punctatosporites*, and *Latosporites*) as well as spores of sub-arborescent *Calamites* (*Calamospora*) and smaller-sized Sphenophyllales. Cordaitaleans are represented by four *Florinites* species, of which *F. mediapudens* is the most abundant (10.6%). Prepollen of other seed plants, such as medullosan prepollen (*Schopfipollenites*), walchian conifer prepollen (*Potoniesporites*), or other pseudosaccates or bisaccates are absent from among the ∼1,500 palynomorphs that have been scanned. The diversity of this sample is 39, and its evenness is 0.76.

In Sample 2, the cordaitalean prepollen *Florinites* dominates the sporomorph assemblage (50.3%), while spores of ferns (28.3%) and arborescent lycopsids (16.0%) are also abundant (Fig. 1). In this sample, *Granasporites medius* is the only abundant spore produced by the Lepidodendrales. Sigillarian lycopsid spores (*Crassispora kosankei*) are common, and spores of herbaceous lycopsids (*Cirratriradites*) are rare in this sample. Marattialean tree ferns are represented by *Cyclogranisporites* (5.3%), and sub-arborescent *Calamites* is represented by *Calamospora*. Spores of other ferns and the smaller-sized Sphenophyllales are present, but in low numbers. Prepollen produced by other seed plants, conifers and seed ferns, is not present in this sample. The diversity of this sample is 32, and its evenness is 0.69.

### Comparison of samples

**Figure 2:**
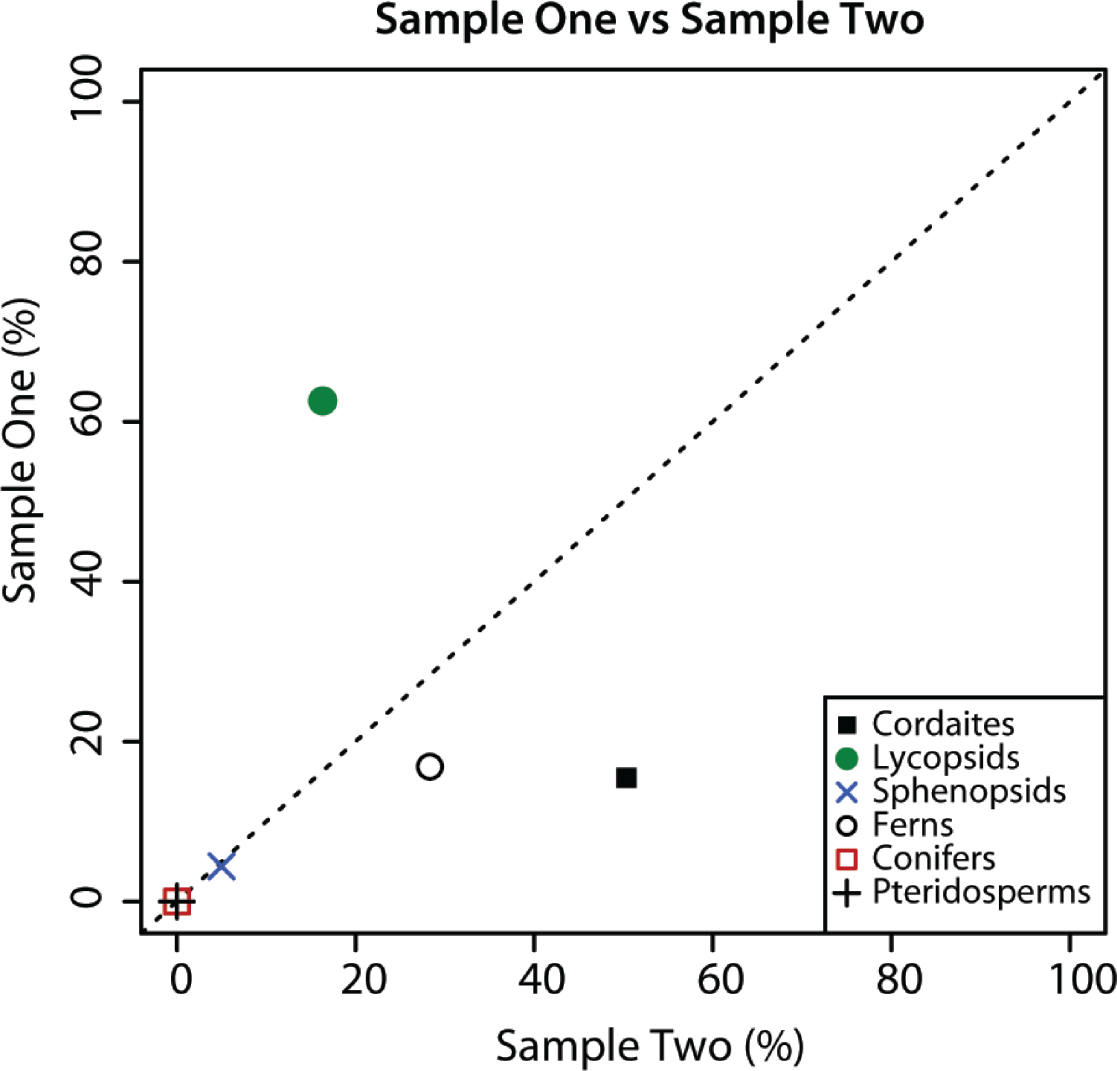
Composition of palynological Sample 1 and 2. Graphical comparison of the composition of palynological Sample 1 and Sample 2 from Cottage Grove plant locality, Cottage Grove Mine, southeast Illinois. Dashed diagonal line represents a line of equality. Major plant groups from Table 2.

The sporomorph assemblage of Samples 1 and 2 are composed of the same plant groups, but the relative abundance of these groups in the two samples is substantially different (Fig. 2). Although sphenopsid spores are a minor component of sporomorph assemblages in Samples 1 and 2, cordaitaleans and ferns are much more abundant in Sample 2 than in Sample 1, and lycopsids are considerably more abundant in Sample 1 than in Sample 2 (Fig. 2). There are also major differences in the species-level composition of the two samples. For example, of the 54 sporomorph species that were recorded in the two samples, just 17 are present in both (Table 1), and a Sorensen’s index comparison of Sample 1 and Sample 2 returns a value of 0.48 (Table 2) (SI = 20C/[A + B], where C is the number of species in common between two samples, and A and B are the total number of species in each of the two samples (McElwain et al., 2007)). These differences are surprising given the stratigraphic proximity of Sample 1 and Sample 2. Sample 2, however, originated from a slightly darker area on the counterpart surface of the same conglomeratic unit, and it is possible that this reflects subtle differences in the taphonomic conditions of the two samples. Factors that could create compositional differences between the two samples include the hydrodynamic regime (Havinga, 1967), the degree of oxidation or microbial activity, and the action of wet and dry cycles (Campbell & Campbell, 1994; see Mander et al., 2012 for a review).

**Figure 3:**
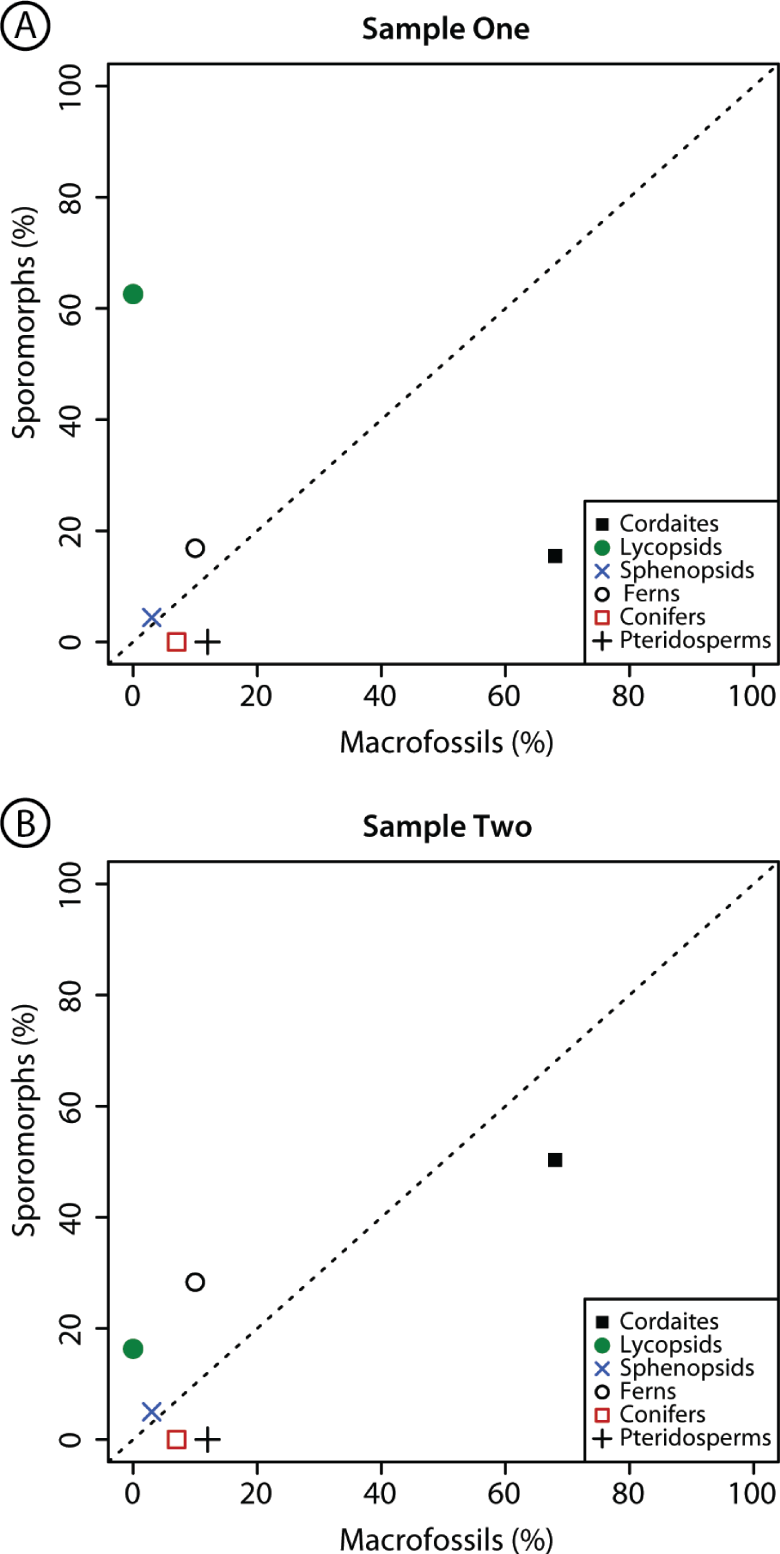
Comparison composition macrofossil and sporomorph assemblages. Graphical comparison of the composition of macrofossil (leaves and other vegetative and reproductive structures) and sporomorph assemblages at Cottage Grove plant locality, Cottage Grove Mine, southeast Illinois. Dashed diagonal line represents a line of equality. Major plant groups from Table 2.

**Table 2:** Abundance major plant groups in macrofossil and sporomorph assemblages. Relative abundance of major plant groups in the macrofossil and sporomorph records at the Cottage Grove plant locality, Cottage Grove Mine, southeast Illinois. See Table 1 for count data.

Plant group	Macrofossils (%)	Sample One (%)	Sample Two (%)
Cordaites	68.3	15.5	50.3
Lycopsids	0.0	62.6	16.3
Sphenopsids	2.4	4.4	5.0
Ferns	9.8	16.9	28.3
Conifers	7.3	0.0	0.0
Pteridosperms	12.2	0.0	0.0

### Comparison of sporomorph and macrofossil assemblages at Cottage Grove

There are considerable differences in the relative abundance of the plant groups that are present as sporomorphs in Samples 1 and 2 and the plant groups that are present as macrofossils at the same locality (Fig. 3; Table 2). Cordaitaleans, dominating the macroflora, are less abundant in the sporomorph record at Cottage Grove. This difference is particularly striking in Sample 1, in which sporomorphs produced by cordaitaleans represent just 15.5% of the total sum (Fig. 3; Table 2). Lycopsid spores are major components of the sporomorph record at Cottage Grove, comprising 62.6% of the total sum in Sample 1, but this plant group is absent from the macrofossil record at this locality (Fig. 3; Table 2). Ferns are also more abundant in the sporomorph record than the macrofossil record at Cottage Grove, while sphenopsids are a minor component of both the sporomorph and macrofossil records at this locality (Fig. 3; Table 2). Walchian conifers and medullosan pteridosperms comprise ∼7% and 12% of the identifiable macrofossils recovered from sediments at Cottage Grove, but prepollen produced by these two plant groups is completely absent from both palynological samples (Fig. 3; Table 2).

## Discussion

This comparison of the macrofossil and sporomorph records at Cottage Grove builds upon previous comparisons of these two fossil groups in the Carboniferous (e.g., Mahaffy, 1988; Willard, 1993; DiMichele & Phillips, 1994), and emphasizes that they provide very different pictures of the standing vegetation at a given locality (e.g., Chaloner, 1968; Gastaldo et al., 1998; Jackson & Booth, 2007; Mander, Kürschner & McElwain, 2010). Especially the absence of prepollen of medulosan pteridosperms (e.g., *Schopfipollenites* and *Monoletes*) and walchian conifers (*Potonieisporites*) in Cottage Grove’s palynological samples is striking. It has been suggested that certain differences between the pictures provided by each fossil group may be explained by palynological processing techniques. For example, large (>200 µm) prepollen referable to medullosan pteridosperms can be inadvertently removed from palynological preparations by sieving over coarse mesh (DiMichele & Phillips, 1994). However, our samples were only sieved over 15 µm mesh, not 200–250 µm mesh as is standard in some palynological processing protocols. Therefore, in contrast to previous suggestions (DiMichele & Phillips, 1994), sieving cannot explain why these plants are present in the macrofossil record at Cottage Grove (Falcon-Lang et al., 2009), but entirely absent from our palynological samples from the same sediments (Fig. 3; Tables 2 and 1). It is possible that our samples, which are derived from very thin siltstone horizons, represent very brief intervals of geological time in which there was no conifer pollen being produced by plants in the source vegetation.

Other differences between the macrofossil and sporomorph records of vegetation at Cottage Grove could be explained by the different ways in which macrofossils and sporomorphs are representative of the standing vegetation, in terms of composition, dominance and spatial scale. In general, macrofossil assemblages are typically weighted towards plants of large stature that produce a considerable number of potential fossils that may disperse widely (e.g., Spicer, 1989; Greenwood, 1991; Gastaldo, 2001). In addition, parautochthonous macrofossil assemblages will tend to be strongly biased toward those plants living in close proximity to the environment of deposition (Scheihing & Pfefferkorn, 1984; Burnham, Wing & Parker, 1992). In contrast, sporomorph assemblages are weighted towards taxa with high sporomorph productivity and/or taxa that produce sporomorphs that are deposited slowly from the atmosphere (Prentice, 1985). Sporomorphs also can be carried longer distances from source areas by water than is typical for foliage, and certain depositional settings, such as large lakes, therefore may have a significant extra-local elements not represented among the macrofossils (Farley, 1990).

In addition to the absence of conifer and pteridosperm prepollen, there is a distinct mismatch between cordaitalean abundance in the Cottage Grove macro and microfloras. The dominance of *Cordaites* leaves in the macrofossil assemblages at Cottage Grove (Falcon-Lang et al., 2009; Fig. 3; Table 2) may be partially a taphonomic bias, reflecting their robust, “leathery” construction (Stewart & Rothwell, 1993, p. 400). Their morphology made them differentially resistant to destruction, particularly if carried in relatively coarse, bed-load sediment. There are suggestions of a counter bias with regard to cordaitalean pollen. Comparison of macrofossil and sporomorph assemblages in Westphalian Coals indicated that sporomorphs produced by cordaitaleans were under-represented relative to macrofossils of the same genus (DiMichele & Phillips, 1994), an observation consistent with our data (Fig. 3; Table 2).

The opposite of the walchian conifer and medullosan pteridosperm pattern is that of the lycopsids. The microfossil-macrofossil mismatch in terms of lycopsid abundance in Cottage Grove Sample 1 (Fig. 3) only partially reflects the high abundance of three *Lycospora* species in sporomoph sample (Table 1). Over-representation of *Lycospora* relative to macrofossil estimates of parent-plant abundances also matches comparisons from Westphalian coals (DiMichele & Phillips, 1994). The relative over-representation of lycopsids in Sample 2 from Cottage Grove (Fig. 3; Table 2), however, is mainly due to the high abundance of *Granasporites* in this sample (Table 1). This is unexpected because *Granasporites* is thought to be under-represented in sporomorph assemblages due to low spore productivity by the parent plants, *Diaphorodendron* and *Synchysidendron* (DiMichele & Phillips, 1994). This may indicate that some results from macrofossil–sporomorph comparisons in peats and coal balls (e.g., DiMichele & Phillips, 1994) cannot be generalized, but it is more likely that there were cryptic populations of *Granisporites* producing plants nearby on the landscape.

### Implications for the dynamics of Pennsylvanian tropical lowland vegetation

Our palynological data indicate that the macrofossil record fails to sample a considerable population of lycopsids at Cottage Grove (Fig. 3; Table 2). This population comprises a total of 17 species (Table 1) and represents between ∼16% (Sample 2) and ∼63% (Sample 1) of the total sporomorph assemblages at this locality (Table 2). As a consequence of the abundance and diversity of this population, we rule out reworking of sporomorphs as a primary cause for the difference between the macrofossil and sporomorph records at Cottage Grove. This population includes *Lycospora granulata*, a spore produced by the highly specialized wetland arborescent lycopsid *Lepidophloios hallii* (DiMichele, 2014), and indicates that wetland plants were present on the seasonally dry landscape near Cottage Grove. A seasonally dry climate at Cottage Grove cannot be inferred from the dominance of cordaitaleans alone, since this group of plants has broad environmental affinities. However, the macrofossil assemblage at Cottage Grove is devoid of lowland wetland plants and contains drought-tolerant walchian conifers (Falcon-Lang et al., 2009). Additionally, the Cottage Grove channel deposit is located within a paleosol interval. It is entirely encased in, and formed lateral to and contemporaneously with a calcic vertisol below the Baker coal bed (Falcon-Lang et al., 2009), which indicates that the climate regime was characterized by sufficient evapotranspiration to result in carbonate deposition within the soil. This paleobotanical and geological evidence together supports the idea that the Pennsylvanian interglacial climate at Cottage Grove was seasonally dry.

The exact geographic location of wetland plants at Cottage Grove cannot be determined from our data. Sporomorphs can be transported considerable distances by wind and water (Prentice, 1985; Sugita, 1993; Hofmann, 2002), so it is possible that they were present in sites some distance from the interfluves on which the local vegetation is thought to have grown (Falcon-Lang et al., 2009). In the context of Pennsylvanian glacial–interglacial cycles (Eros et al., 2012), we suggest that the wetland taxa in our palynological analysis represent the survival of these plants in refugia during the seasonally dry parts of glacial–interglacial cycles. This supports the idea of the Pennsylvanian Coal Forest as a dynamic biome expanding, contracting and fragmenting in concert with changes in the prevailing climate (Falcon-Lang & DiMichele, 2010; DiMichele, 2014). Such refugia are likely to have been spatially discontinuous, small, wet areas such as inland swamps, waterside habitats, and coastal wetlands (DiMichele, 2014), or dry season waterholes (Bashforth et al., 2014).

## Supplemental Information

10.7717/peerj.630/supp-1 Table S1 Sporomorph count data sample 1 and 2, Cottage Grove plant locality, Cottage Grove Mine, southeast Illinois.
